# Fused *eco29kIR- *and *M *genes coding for a fully functional hybrid polypeptide as a model of molecular evolution of restriction-modification systems

**DOI:** 10.1186/1471-2148-11-35

**Published:** 2011-02-03

**Authors:** Marina L Mokrishcheva, Alexander S Solonin, Dmitri V Nikitin

**Affiliations:** 1 Institute of Biochemistry and Physiology of Microorganisms, Russian Academy of Sciences, Prospekt Nauki, 5, Pushchino, Moscow region 142290, Russia; 2 Pushchino State University, Prospekt Nauki, 3, Pushchino, Moscow region 142290, Russia

## Abstract

**Background:**

The discovery of restriction endonucleases and modification DNA methyltransferases, key instruments of genetic engineering, opened a new era of molecular biology through development of the recombinant DNA technology. Today, the number of potential proteins assigned to type II restriction enzymes alone is beyond 6000, which probably reflects the high diversity of evolutionary pathways. Here we present experimental evidence that a new type IIC restriction and modification enzymes carrying both activities in a single polypeptide could result from fusion of the appropriate genes from preexisting bipartite restriction-modification systems.

**Results:**

Fusion of *eco29kIR *and *M *ORFs gave a novel gene encoding for a fully functional hybrid polypeptide that carried both restriction endonuclease and DNA methyltransferase activities. It has been placed into a subclass of type II restriction and modification enzymes - type IIC. Its MTase activity, 80% that of the M.Eco29kI enzyme, remained almost unchanged, while its REase activity decreased by three times, concurrently with changed reaction optima, which presumably can be caused by increased steric hindrance in interaction with the substrate. *In vitro *the enzyme preferentially cuts DNA, with only a low level of DNA modification detected. *In vivo *new RMS can provide a 10^2^-fold less protection of host cells against phage invasion.

**Conclusions:**

We propose a molecular mechanism of appearing of type IIC restriction-modification and M.SsoII-related enzymes, as well as other multifunctional proteins. As shown, gene fusion could play an important role in evolution of restriction-modification systems and be responsible for the enzyme subclass interconversion. Based on the proposed approach, hundreds of new type IIC enzymes can be generated using head-to-tail oriented type I, II, and III restriction and modification genes. These bifunctional polypeptides can serve a basis for enzymes with altered recognition specificities. Lastly, this study demonstrates that protein fusion may change biochemical properties of the involved enzymes, thus giving a starting point for their further evolutionary divergence.

## Background

DNA restriction-modification systems (RMS) are prokaryotic tools against invasion of foreign DNAs into cells [1]. They play an important evolutionary role as subcellular barriers restricting horizontal gene transfer and thereby providing microbial biodiversity. Usually, RMS comprise of a restriction endonuclease (REase) and modification DNA methyltransferase (MTase) enzyme recognizing the same short 4-8 nucleotide sequence. RMS functioning includes methylation of recognition DNA sequences by MTase. All non-modified sites can be cut by a cognate REase [1]. Type II REases are indispensable tools in creating recombinant DNA molecules [2]. Their widespread practical application has stimulated research to discover and characterize more of these systems. Currently, more than 6000 different sequences corresponding to REases of type II alone are listed in REBASE, the database holding all known and many putative RMS [3].

The high number of known RMS is reflected also in high diversity of their organization or functioning and, hypothetically, in multiplicity of their evolutionary pathways. One of these pathways could be fusion of preexisting ORFs with formation of a gene capable of producing a protein with an array of new activities and functions. It could be suggested that type IIC RMS carrying both REase and MTase in a single polypeptide might appear by this mechanism [4]. Here we report direct evidence how a fully functional type IIC REase could appear by fusion of the appropriate genes as a result of a few point mutations.

**Figure 1:**
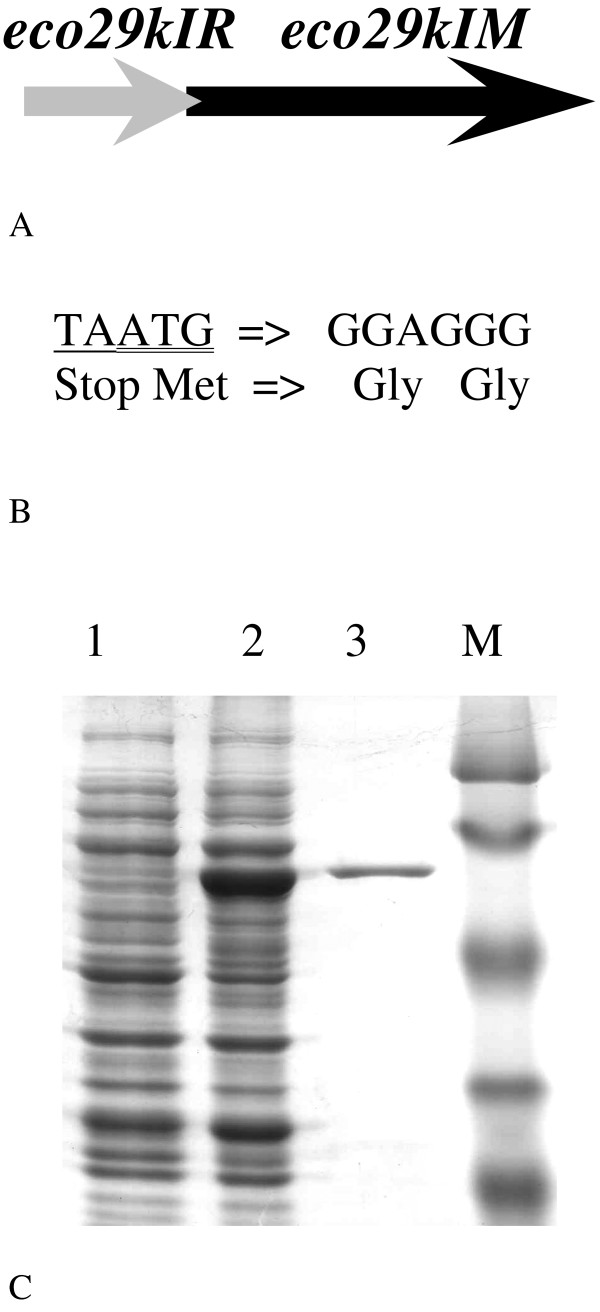
**Overproducing strain construction and purification of protein RM.Eco29kI**. A - *eco29kIR *and *M *ORFs orientation on the natural plasmid pECO29 [5]. On the plasmid Stop codon of *eco29kIR *gene overlaps Start codon of *eco29kIM *gene. B - A scheme of site-directed mutagenesis used for generation of the fusion protein RM.Eco29kI. Overlapped Stop and Start codons were substitutes for two Glycine codons. C - 10% PAGE electrophoresis with RM.Eco29kI induction (lane 2); final preparation of the enzyme (lane 3). Lane 1 - protein extract from non-induced cells, M - Dalton markers of 26, 34, 47, 86 and 120 kDA.

As an object of our experiment Eco29kI RMS was chosen. This RMS is carried by the natural plasmid pECO29 found in clinical *E. coli *29kI isolate [5]. On this plasmid there were genes coding for 214 aa REase and 382 aa DNA MTase [6,7]. By nomenclature Eco29kI RMS belongs to IIP group as one recognizing the palindromic site [4]. Its REase contains GIY-YIG nuclease domain that was identified in homing endonucleases, DNA repair and recombination enzymes, and restriction endonucleases [8,9]. Recently its mechanism of action was established: R.Eco29kI monomers dimerize on a single cognate DNA molecule forming the catalytically active complex [10]. An Eco29kI RMS is organized in such a way that REase ORF precedes MTase ORF, and the REase Stop codon and the MTase Start codon overlap (Figure 1A and 1B). Similar organization is also characteristic to such well-known RMS as SalI and HindIII. Using site-directed mutagenesis, *eco29kI R *and *M *ORFs were fused to give a fully functional hybrid protein. It was purified and characterized. Its biochemical properties, namely, REase and MTase specificities and the optima of reaction conditions were compared with those of the original enzymes.

Apparently, a similar mechanism of gene fusion via natural point mutations/deletions/insertions/invertions/translocations could give type IIC REases as well as bifunctional MTases like SsoII-related enzymes and bifunctional MTases like FokI and LlaI [4,11,12]. Apart from methyltransferase, these proteins also possess separate domains involved in transcriptional regulation (SsoII) or methylation (FokI and LlaI). It should be noted that gene fusion is facilitated by the head-to-tail gene orientation, which is quite common for many RMS. For example, the following type II RM systems are organized in this way: AccI, BanI, Bsp6I, BsuBI, Cfr9I, DdeI, EagI, EcoPI, EcoP15, EcoRI, FnuDI, HaeIII, HgiBI, HgiCI, HgiCII, HgiDI, HgiEI, HgiGI, HhaII, HincII, HindIII, HinfI, HpaI, MboII, MwoI, NcoI, NdeI, NgoMI, NgoPII, NlaIII, PaeR7I, RsrI, SalI, Sau3A, Sau96I, TaqI, TthHB8I, XbaI, and XmaI, and many more can be found in RMS database [3,12]. Thus, their unidentified type IIC bifunctional derivatives could exist in nature.

## Results

### Construction of overproducing strain and purification of RM.Eco29kI

To construct an overproducing strain for the protein RM.Eco29kI, ORFs of Eco29kI REase and MTase were amplified from the natural plasmid pECO29 where they are oriented as shown in Figure 1A[5],. On this plasmid the Stop codon of Eco29kI REase and the Start codon of Eco29kI MTase overlap. Subsequently they were cloned in the same orientation into the pET19mod vector. By site-directed mutagenesis their separating Stop and Start codons were substituted for 2 Glycine codons, as shown in Figure 1B, thus forming the *eco29kI.RM *gene. The resulting RM.Eco29kI polypeptide contains both REase and MTase enzymes joint by a flexible 2 Glycine hinge and 6 His-tag on its N terminus.

The purification scheme for RM.Eco29kI was based on affinity (Ni-CAM, nickel chelate affinity matrix, Sigma) chromatography. From a Ni-CAM column RM.Eco29kI was eluted by linear steps of 20, 50, 75, 100 and 150 mM imidazole. Finally, 200 ml of cell culture gave about 0.4 mg of >98% purified enzyme (Figure 1C) with a molecular weight of ~67 kDa.

When the Stop codon of Eco29kI REase gene was substituted for Glycine without modifying the first Methionine codon of Eco29kI MTase gene, expression of M.Eco29kI, but not RM.Eco29kI, was detected (unpublished observations). Hypothetically, it might happen due to a pronounced secondary structure of mRNA in the region corresponding to R.Eco29kI ORF or, as it was reported personally by M. Nagornykh, to transcription initiation from an alternative promoter closely preceding M.Eco29kI ORF [13].

### Characterization of RM.Eco29kI REase activity

**Figure 2:**
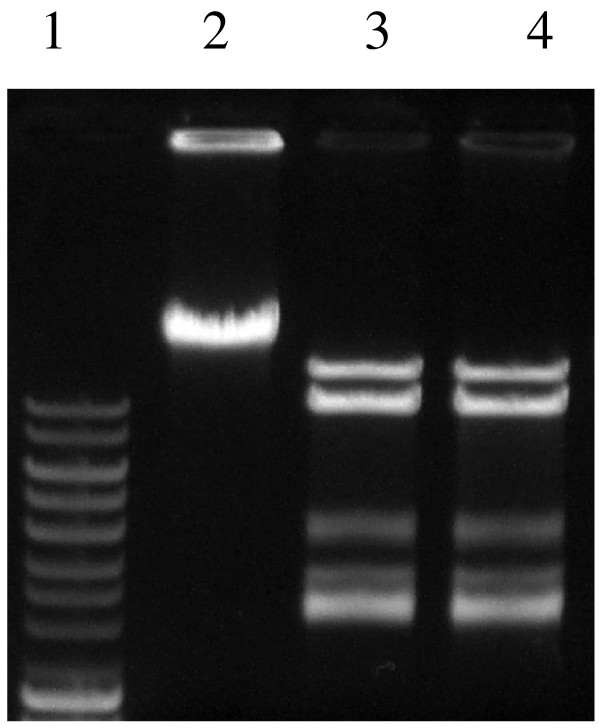
**Agarose gel visualization of the phage φ80vir DNA hydrolysis pattern with RM.Eco29kI (lane 3) and R.Eco29kI (lane 4)**. Lane 1 - Dalton markers: 1000, 1500, 2000, 2500, 3000, 4000, 5000, 6000, 8000, and 10000 bp; lane 2 - uncut phage φ80vir DNA.

Two enzymes, R.Eco29kI and RM.Eco29kI, purified to homogeneity, were assayed for their recognition specificity and catalytic reaction optimum at varied concentrations of NaCl, KCl, temperature and pH. R.Eco29kI was purified as described previously [14]. Figure 2 shows hydrolysis patterns of φ80vir DNA with R.Eco29kI (lane 4) and with RM.Eco29kI (lane 3). The patterns are the same, so the hybrid protein RM.Eco29kI retained the specificity of R.Eco29kI.

**Figure 3:**
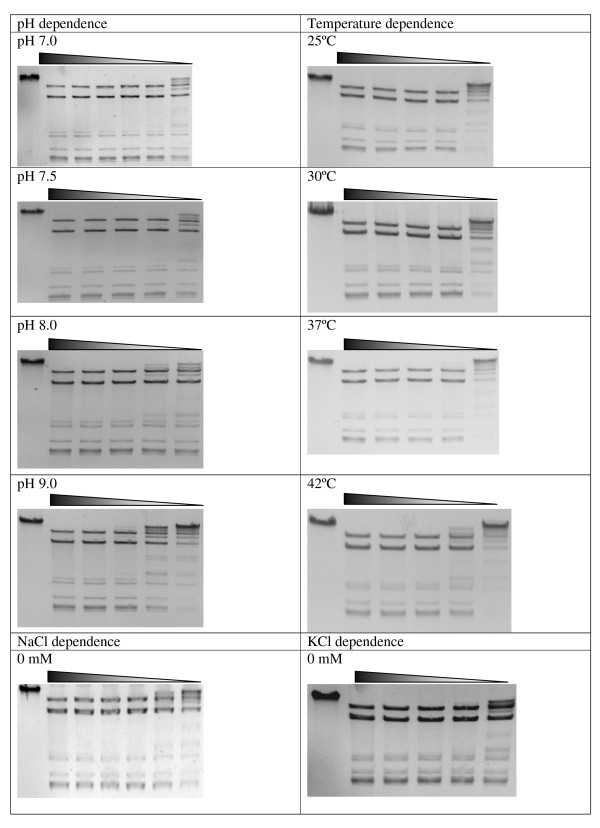
**Reaction optima characterization for RM.Eco29kI**. Triangles above gels mark 2-fold dilutions of the enzyme in the reaction mixtures. First lane of each gel represents uncut phage φ80vir DNA. The gels represent RM.Eco29kI activity depending on pH, temperature, NaCl and KCl concentrations. Varied parameters are written above each gel.

**Figure 4:**
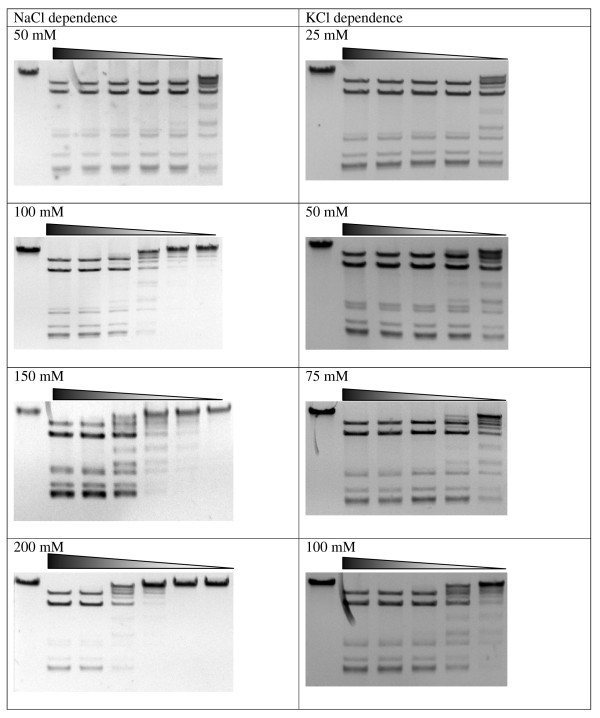
**Reaction optima characterization for RM.Eco29kI, continuation of Figure 3**.

Then the enzyme RM.Eco29kI was assayed for catalytic reaction optimum. The maximal catalytic REase activity of the hybrid protein was observed at 0-50 mM NaCl; 0-25 mM KCl, 10 mM MgCl_2_; pH 7.0, at 30-37°C (Figure 3 and 4). Table 1 presents reaction optimum comparison for R.Eco29kI and RM.Eco29kI. As seen, the optima were slightly changed: 100 mM less for NaCl, 50 mM less for KCl, and 1 unit less for pH. It means that after fusion the biochemical properties of the REase part of the protein were changed, despite its intact amino acid sequence. Under optimal conditions R.Eco29kI had a specific activity of 60 AU/pMol, whereas RM.Eco29kI had 20 AU/pMol amounting to 33% of the native value. Thus, after fusion with M.Eco29kI its activity was decreased by about 3 times.

**Table 1:** Comparison of reaction optima for R.Eco29kI and fused RM.Eco29kI.

	**R.Eco29kI **[14]	RM.Eco29kI
[NaCl], mM	100-150	0-50
[KCl], mM	25-75	0-25
pH	7.0-8.0	7.0
T, °C	30-37	30-37

### Characterization of RM.Eco29kI MTase activity

The biochemical characterization of Eco29kI MTase is presented in [15]. The enzyme methylates the second Cytosine in the sequence C**C^Me^**GCGG. Specificity of RM.Eco29kI MTase activity was proved to be the same, because there was no incorporation of labeled methyl groups into substrates pretreated with M.Eco29kI enzyme and non-labeled AdoMet. The optima of reaction conditions also remained unchanged: both enzymes showed their maximal activities at 50 mM NaCl; 5 mM EDTA; pH 7.0-8.5, and 37°C. Under optimal conditions M.Eco29kI had a specific activity of 10 AU/pMol, whereas RM.Eco29kI had 8 AU/pMol amounting to 80% of the native value. Thus, after fusion with R.Eco29kI, activity of its MTase part was almost unchanged.

### Characterization of RM.Eco29kI functioning *in vivo*

**Figure 5:**
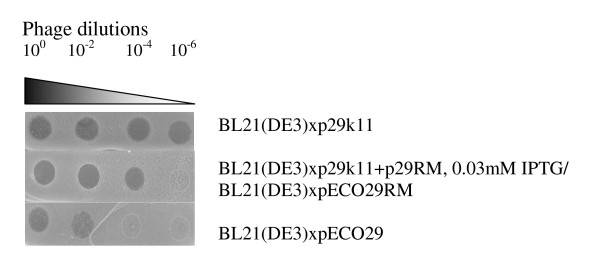
**Phage restrictions by RM.Eco29k1**. Triangle marks phage dilutions which are shown above. BL21(DE3)xp29k11 cells carry only gene coding for Eco29kI MTase and lack Eco29kI REase activity. BL21(DE3)xpECO29 cells carry natural pECO29 plasmid, having both MTase and REase activities of the wild type Eco29kI RMS. BL21(DE3)xp29k11+p29RM cells carry genes coding for M.Eco29kI and RM.Eco29kI enzymes on two different plasmids.

To characterize *in vivo *functioning of new RMS, we performed phage restriction experiments. In these experiments 100-fold dilutions of phage λvir (10^0^, 10^-2^, 10^-4^, 10^-6^) were spotted on lawns of bacterial cells. The results are presented in Figure 5. BL21(DE3)xp29k11 cells carry only gene coding for Eco29kI MTase and lack Eco29kI REase activity, which allows evaluating total concentration of infective phage λvir virions. BL21(DE3)xpECO29 cells carry natural pECO29 plasmid, having both MTase and REase activities of the wild type Eco29kI RMS. BL21(DE3)xpECO29RM cells carry gene coding for RM.Eco29kI enzyme on the natural pECO29 plasmid. BL21(DE3)xp29k11+p29RM cells carry genes coding for M.Eco29kI and RM.Eco29kI enzymes. As seen from the figure, RM.Eco29kI induction by low concentration of IPTG (0.03 mM) led to a 10^2^-fold cell protection against phage invasions, so that only one out of 100 virions could infect the cells. The same values were obtained for RM.Eco29kI enzyme carried on natural pECO29 plasmid (pECO29RM variant). Under the given conditions wild type Eco29kI RMS protected cells more effectively, giving a 10^4^-fold restriction, so that only one out of 10, 000 virions could infect the cells. Thus, the new RMS protected its host cells 10^2 ^times less effectively than the wild type RMS. But 10^2 ^phage restriction value reflects the ability of the new RMS to protect cells against foreign DNA invasion; so this function of the wild type RMS was conserved in the RM.Eco29kI RMS.

### Characterization of RM.Eco29kI behavior *in vitro*

**Figure 6:**
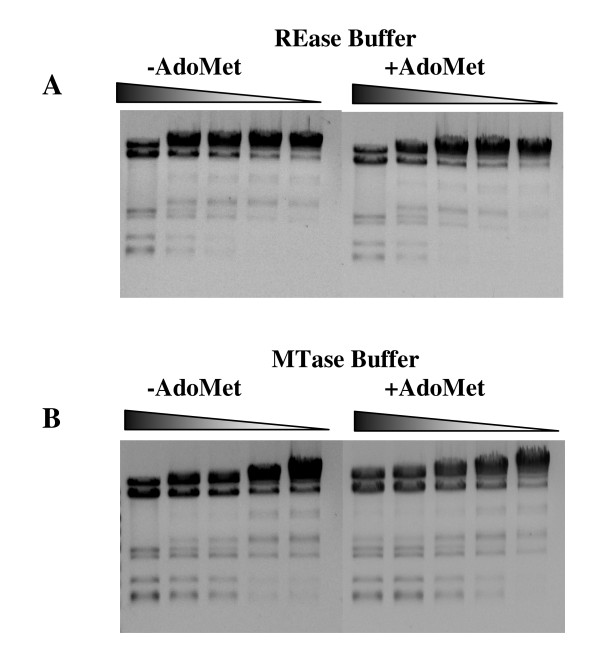
**Analysis of the AdoMet effect on REase activity of RM.Eco29kI**. A - reactions were carried out in REase optimal buffer in the absence (left panel, -AdoMet) and presence (right panel, +AdoMet) of AdoMet; B - reactions were carried out in MTase optimal buffer in the absence (left panel, -AdoMet) and presence (right panel, +AdoMet) of AdoMet. Triangles above gels mark 2-fold dilutions of the enzyme in the reaction mixtures. The figure represents ratio of REase and MTase activities in RM.Eco29kI enzyme.

To assess the *in vitro *interaction of the bifunctional enzyme RM.Eco29kI with DNA and the effect of AdoMet on its REase activity, we incubated this protein with phage φ80vir DNA in conditions optimal for REase (Figure 6A) and MTase (Figure 6B) in the absence or presence of excess AdoMet (10 μM). In MTase reaction mixture we substituted 5 mM EDTA for 10 mM MgCl_2 _to supply magnesium ions to the REase part of the enzyme. While in REase buffer the hydrolysis patterns looked identical both in the presence and absence of AdoMet, in MTase buffer with AdoMet a slightly incomplete hydrolysis could be observed even at the lowest enzyme dilutions (Figure 6B), which could be explained by DNA methylation with the MTase part of the enzyme. It follows from these results that *in vitro *RM.Eco29kI enzymatic reaction is strongly biased towards DNA hydrolysis, while only a small portion of DNA can be modified in the same reaction mixture; and that AdoMet does not influence REase activity of the enzyme, unlike other type IIC proteins known so far.

## Discussion

### Nomenclature of the RM.Eco29kI enzyme

To date, the following 12 proteins have been proved to show both REase and MTase activities by one polypeptide chain: AloI, BcgI, BseMII, BseRI, BsgI, BspLU11III, CjeI, Eco57I, HaeIV, MmeI, PpiI, and TstI [16-26]. They can be considered as members of the type IIC RMS group. Their properties are given in Table 2. All of them also belong to IIB (cutting on both sides of their recognition sequences) or to IIG (stimulated or inhibited with AdoMet) groups of RMS [4]. The RM.Eco29kI protein also falls in the category of IIC enzymes, but differs from its regular members by many features. It recognizes a true non-interrupted palindromic sequence; it cuts within the recognition site; its REase is not influenced by AdoMet; and its MTase belongs to m^5^C type, while others contain m^6^A type MTases. Altogether, properties of RM.Eco29kI expand the limits of type IIC enzymes.

**Table 2:** Major properties of proved type IIC enzymes.

Enzyme	Other subtypes	Recognition sequence	AdoMet influence on REase	Type of MTase	Presence of separate M gene
AloI [16]	B, G, S	(7/12-13) GAACNNNNNNTCC (12-13/7)	yes	m^6^A	no
BcgI [17]	B, G, H, S	(10/12) CGANNNNNNTGC (12/10)	yes	m^6^A	no
BseMII [18]	G, S	CTCAG (10/8)	yes	m^6^A	yes
BseRI [19]	G, S	GAGGAG (10/8)	yes	m^6^A	no
BsgI [20]	G, S	GTGCAG (16/14)	yes	m^6^A	no
BspLU11III [21]	G, S	GGGAC (10/14)	yes	m^6^A	yes, two genes
CjeI [22]	B, G, S	(8/14) CCANNNNNNGT (15/9)	yes	m^6^A	no
RM.Eco29kI This report	P	CCGC^GG	no	m^5^C	yes
Eco57I [23]	E, G, S	CTGAAG (16/14)	yes	m^6^A	yes
HaeIV [24]	B, G, P	(7/13) GAYNNNNNRTC (14/9)	yes	m^6^A	no
MmeI [25]	G, S	TCCRAC (20/18) or TCCRAC (21/19)	yes	m^6^A	no
PpiI [26]	B, G, S	(7/12) GAACNNNNNCTC (13/8)	yes	m^6^A	no
TstI [26]	B, G, S	(8/13) CACNNNNNNTCC (12/7)	yes	m^6^A	no

### The role of gene fusion in molecular evolution of RMS

In our study a fully functional hybrid polypeptide was generated by fusion of Eco29kI REase and MTase proteins. New protein had REase and MTase specificities of the original enzymes. Its MTase activity was almost unchanged and amounted to 80% of that of the M.Eco29kI under optimal reaction conditions for both enzymes: 50 mM NaCl; 5 mM EDTA; pH 8.0, and 37°C [15]. Its REase activity was decreased by three times, which could be attributed to increased steric hindrance in interaction with substrate. Besides, the reaction optimum for REase activity of RM.Eco29kI differed from that of the R.Eco29kI as follows: 100 mM less for [NaCl], 50 mM less for [KCl], and 1 unit less for pH (Table 1). The particular reason for this shift is unclear because many physical and chemical properties of the protein, such as molecular weight, isoelectric point, total charge, geometry of the protein, surface charge distribution, etc., were different from those of the original proteins. Hypothetically, the gap between their properties could yield as a result of the natural selection, after many generations, a novel protein with altered biochemical properties and different functions in the cell.

**Figure 7:**
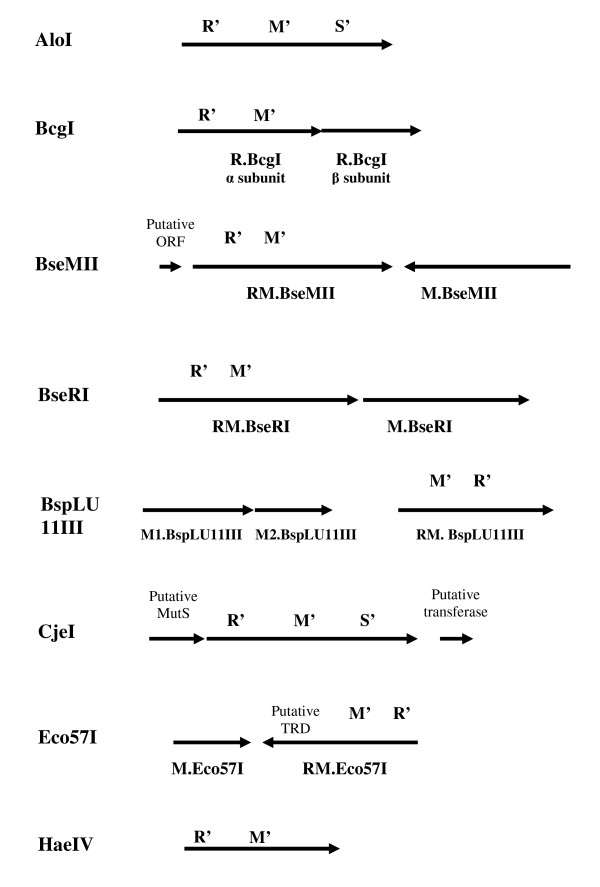
**Organization schemes of proved type IIC RMS**. Black arrows show localization of corresponding ORFs. R' - REase part; M' - MTase part; S' - DNA recognition parts of enzymes. The figure demonstrates gene organization of characterized type IIC RMS.

**Figure 8:**
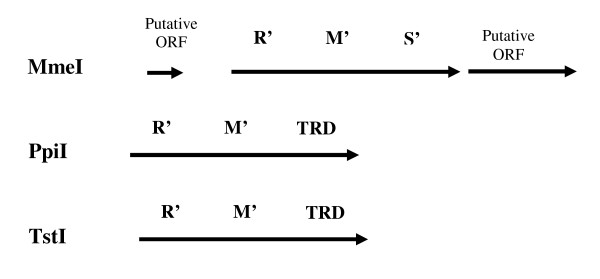
**Organization schemes of proved type IIC RMS, continuation of Figure 7**.

This work presents experimental evidence for molecular evolution of RMS or multifunctional proteins in general. It has been directly shown that a few point mutations can result in a protein with a novel combination of activities and altered properties. On the one hand, it may be considered as a molecular mechanism for appearance of type IIC RMS enzymes. Figure 7 and 8 show a schematic gene organization of the described type IIC RMS. As seen, all of them could result from fusion of head-to-tail oriented endonuclease and methyltransferase ORFs. Possible involvement of gene fusion was proposed for the formation of AloI, BseMII, CjeI, Eco57I, HaeIV, and MmeI. The enzymes AloI, CjeI, and HaeIV probably resulted from fusion of HsdS, HsdM and REase domains; Eco57I - from Mod and Res subunits of type III enzymes; while BseMII and MmeI - from REase and MTase domains [16,18,23,25].

It can be predicted that novel type IIC RMS may appear via fusion of genes from bipartite RMS having head-to-tail gene organization. This type of gene organization is quite common for RMS of all types. Type I RMS such as CfrA, EcoA, EcoB, EcoD, EcoE, EcoK, EcoR124, StySB, StySI, StySP, and StySQ; type II RMS such as AccI, BanI, Bsp6I, BsuBI, Cfr9I, DdeI, EagI, EcoPI, EcoP15, EcoRI, FnuDI, HaeIII, HgiBI, HgiCI, HgiCII, HgiDI, HgiEI, HgiGI, HhaII, HincII, HindIII, HinfI, HpaI, MboII, MwoI, NcoI, NdeI, NgoMI, NgoPII, NlaIII, PaeR7I, RsrI, SalI, Sau3A, Sau96I, TaqI, TthHB8I, XbaI, and XmaI, all have head-to-tail gene orientation, thus being good candidates for originating new type IIC enzymes [12]. These possibilities could be facilitated by increased affinity of DNA MTases for hemimethylated substrates in comparison with non-methylated ones, unlike their REase counterparts. Therefore, if a fused RMS appeared from preexisting bipartite RMS and its MTase activity was lower of the original enzyme, its propagation would be facilitated by facts that all recognition sites in the host genome were methylated or, after replication, hemimethylated by the preexisting enzyme and that it had increased affinity to hemimethylated substrates. Otherwise, decreased MTase activity of a new fused RMS could lead to appearance of unprotected recognition sequences in host genome, which will be cut by REase, host cells will die and this RMS will not propagate in a bacterial population.

**Figure 9:**
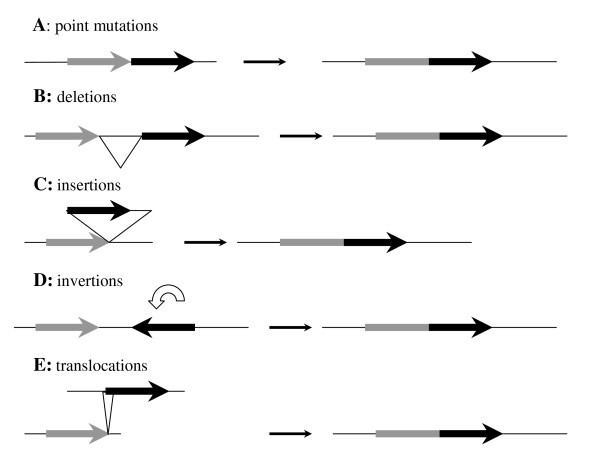
**A scheme of genetic rearrangement events that could lead to single ORF fusions**. Grey and black arrows show any hypothetical ORFs; white circle arrow - inversion of DNA; triangles - recombination between different pieces of DNA. On the figure are shown genetic pathways that could lead to gene fusion.

**Figure 10:**
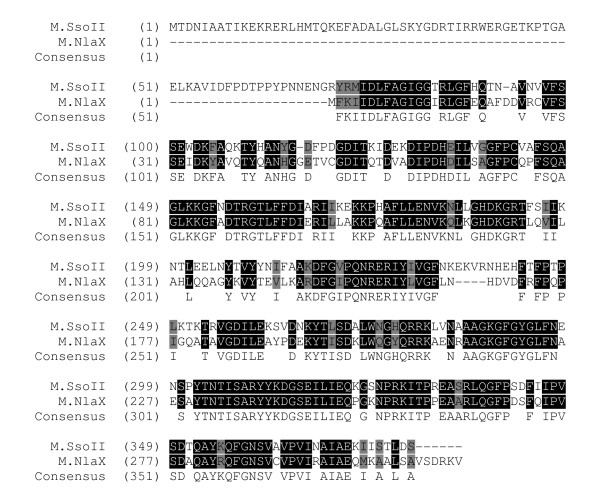
**A scheme of alignment for M.SsoII and M.NlaX**. Identical amino acids are marked by black and similar amino acids by grey background. The figure demonstrates presence of 70 aa N-terminal domain in M.SsoII, but not in M.NlaX.

On the other hand, this study provides an example of a more general mechanism for gaining new functions by existing proteins. Hypothetically, any pair of ORFs can be joint in-frame by point mutations/deletions/insertions/inversions/translocations or their combinations (Figure 9). Then the newly generated polypeptide may serve as an evolutionary intermediate for the natural selection in improving old or accommodating new functions in the cell. For example, M.SsoII-related bifunctional enzymes, including its izoschizomers, with regulatory and MTase domains could arise by natural fusion of the appropriate ORFs at some stages of their evolutionary history [11,27]. The latter suggestion is supported by the presence of NlaX MTase, a close homolog of M.SsoII without the regulatory N-terminal domain. As shown in Figure 10, both polypeptides display high identity after 70 amino acids of the M.SsoII N-terminal domain known to be involved in its gene autoregulation [11]. Similar fusion of preexisting head-to-tail oriented ORFs coding for C-protein and endonuclease in BamHI, Eco72I, MunI, PvuII, SmaI RMS, could give REases with transcription regulatory functions. Depending on gene organization, there are also possibilities for fusion of two MTases from preexisting RMS, such as DpnII and HgaI, leading to bifunctional MTases like FokI and LlaI [12].

In principle, any other adjacent gene of appropriate orientation could be fused with the REase or MTase part of RMS. In this case the fact that RMS elimination is lethal for cells would defend the fused ORF from being lost [28]. It occurs because long-lasting endonucleolitic activity results in multiple cuts on non-protected genomic DNA, thereby killing cells that lack RMS. Thus, joining RMS genes would promote maintenance of a fused ORF and its spreading in bacterial populations, provided their functioning is not disturbed.

### Practical applications of gene fusion for generating REases with novel recognition sites

Restriction enzymes are robust tools for the recombinant DNA technology. Despite the fact that more than 200 enzymes with different recognition sites have been isolated from various bacterial strains, many specificities have not been discovered [23,26]. To create enzymes with altered recognition specificities, methylation activity-based selection (MABS) and target recognition domain (TRD) reassortment [23,26] approaches were proposed. Using these techniques, bifunctional enzymes of type IIC such as Eco57I, AloI, PpiI, and TstI were manipulated to yield a generation with novel specificities. Most importantly, both of their activities operated on the same target sequence, thus providing a possibility to use the DNA-modification activity of these enzymes for the selection of mutants with altered sequence specificity. To enlarge the list of enzymes available for these manipulations, a gene fusion approach, similar to one used in this work, could be applied. By this procedure, hundreds of bifunctional enzymes could be created, e.g., from type I and III head-to-tail oriented RMS, thus giving an opportunity of significant contribution to the existing recognition specificities.

## Conclusions

Altogether, our work presents an example of molecular mechanism for appearance of type IIC restriction-modification and SsoII MTase-related enzymes as well as other multifunctional proteins. It demonstrates that gene fusion could play an important role in evolution of restriction-modification systems and be responsible for enzyme subclass interconversion. Based on the proposed approach, hundreds of novel type IIC enzymes could be generated from head-to-tail oriented type I, II and III restriction and modification genes. These new bifunctional polypeptides could be useful for creating enzymes with altered recognition specificities. Lastly, our work shows that protein fusion can change biochemical properties of the involved enzymes, thus giving a starting point for their further evolutionary divergence, which, after many generations, gives a novel protein with both altered biochemical properties and different functions in the cell.

## Methods

### Bacterial Culture

All strains used were grown in LB liquid medium with high aeration. For selection ampicillin (100 μg/ml) and kanamycin (25 μg/ml) were added to LB agar plates [29].

### DNA Manipulations

All genetic engineering methods were performed as described [29]. Selection of transformants with plasmids carrying Eco29kI RMS genes and screening of recombinant clones were performed as elsewhere [30,31].

### Chemicals, Bacterial Strains, Plasmids and Enzymes

*E. coli *BL21(DE3) (Novagen) and Z-85 strains were used for transformation experiments [32]. Plasmids pET19mod, pECO29, and p29k11 were used for a number of experiments [5,7,33]. Restriction endonucleases, T4 DNA ligase, and thermostable DNA-polymerase from *Th. aquaticus *were purified in our laboratory. *Pfu *DNA polymerase was purchased from New England Biolabs (UK). Protein and DNA ladders were obtained from Fermentas (Lithuania).

Strain BL21(DE3) (Novagen), transformed with plasmid p29k11 (Km^r^) bearing *eco29kIM *gene was used as a host for transformation of plasmid containing *eco29kI RM *fusion gene [7].

(^3^H-methyl)-labeled AdoMet (15Ci/mMol) was purchased from Amersham Biosciences; unlabeled AdoMet, potassium phosphate, sodium chloride, potassium chloride, bovine serum albumin were from Sigma. Acrylamide, methylenebisacrylamide, Coomassie Brilliant Blue R-250 and Low Melting Agarose (LMP) were from BioRad. All other chemical reagents used were of the highest grade purity.

### Construction of Eco29kI REase and MTase fusion RM.Eco29kI protein overproducing strain and biomass production

Eco29kI REase and MTase genes were amplified from the natural plasmid pECO29 with primers TTTGTCGAC**ATG**CACAATAAGAAATTTGATA (forward; containing start ATG codon shown in bold) and CCTGGATCCCTT**TTA**ATTGAAGTTAGAGCACAA (reverse; containing stop TTA codon shown in bold), carrying SalI and BamHI sites, respectively, for subsequent cloning [5]. Appropriately digested PCR product was cloned into pET19mod vector. Site-directed mutagenesis has been performed to fuse Eco29kI REase and MTase ORFs, using the high fidelity *Pfu *DNA polymerase and oligonucleotide primers:

Fus1: AAGAGTAATTTTACAGGAGGGAGATCATTAGAG and

Fus2: CTCTAATGATCTCCCTCCTGTAAAATTACTCTT.

A novel plasmid containing staggered nicks was generated. Following the thermal cycling, the reaction mixture was treated with DpnI REase that digests hemimethylated parental DNA, leaving behind the newly amplified nicked DNA with the mutation of interest. The mutation and *eco29kI.RM *gene were confirmed by sequencing. The resulting construct was referred to as p29RM.

The plasmid p29RM was introduced into *E. coli *BL21(DE3) strain. Cells were grown in LB medium supplemented with ampicillin and kanamycin at 20°C. After reaching OD_590 _= 0.6, the recombinant protein RM.Eco29kI synthesis was induced by addition of isopropyl-β-D-thiogalactopyranoside (IPTG) to a final concentration of 0.1 mM followed by overnight incubation at 20°C. Cells were harvested by centrifugation, frozen and stored at -70°C. All subsequent steps were carried out at 4°C.

### Construction of natural pECO29 plasmid carrying fused Eco29kI REase and MTase genes

To make Eco29kI REase and MTase gene fusion on natural pECO29 plasmid, its BclI-PvuII fragment was substituted for BclI-PvuII fragment of p29RM plasmid, containing the gene fusion mutations. Resulting plasmid was referred as pECO29RM.

### Protein Purification

RM.Eco29kI-producing *E.coli *cells pelleted from 200 ml of culture were lysed by lysozyme treatment (1 mg/ml, 30 min at 4°C) in 5 ml of ice-cold buffer A (50 mM Tris-HCl, pH 7.5, 300 mM NaCl, 5 mM β-mercaptoethanol, 10 mM Imidazole, and 5% glycerol (v/v)) with protease inhibitor 1 mM phenylmethylsulfonyl fluoride (PMSF), followed by sonication. Triton X-100 was added to the lysate to a final concentration of 1%, and the lysate was subjected to centrifugation at 14,000 rpm, 4°C for 40 min to eliminate cell debris. The resulting supernatant was applied to a Ni-CAM (Sigma) column equilibrated with buffer A with 1% Triton X-100. After washing the column with buffer B (50 mM Tris-HCl, pH 7.5, 1 M NaCl, 5 mM β-mercaptoethanol, 15 mM Imidazole, 5% glycerol (v/v), and 1% Triton X-100), the protein was eluted using linear steps (20-150 mM) of imidazole in buffer A with 0.1% Triton X-100. Active fractions with specific REase activity containing homogenous RM.Eco29kI (as judged by SDS-PAGE) were pooled and dialyzed overnight against storage buffer (50 mM Tris-HCl, pH 7.5, 250 mM NaCl, 7 mM β-mercaptoethanol, 0.1 mM EDTA, 0.1% Triton X-100, 50% glycerol (v/v)) and stored at -20°C. The purified protein was analyzed by SDS-PAGE according to the method of Laemmli using Coumassie Blue R-250 (BioRad, USA) conventional staining [34].

### Characterization of phage restriction by RM.Eco29kI RMS

To characterize RM.Eco29kI RMS ability to protect host cells against phage invasion different dilutions of phage λvir (10^0^, 10^-2^, 10^-4^, 10^-6^) were spotted on bacterial lawns of BL21(DE3)xp29k11 (negative control), BL21(DE3)xp29k11+p29RM (with 0.03 mM IPTG), BL21(DE3)xpECO29RM (*eco29kIRM *gene under natural promoter) and BL21(DE3)xpECO29 (positive control) cells. Then minimal concentrations of phage λvir, capable to infect corresponding strains, were compared.

### Methylation Assay

To test RM.Eco29kI DNA methyltransferase activity, we used DE-filters assay according to [35].

### Analysis of Protein Concentration

Protein concentration was determined from the absorption at 280 nm on a Shimadzu UV-1601 spectrophotometer (Japan). An extinction coefficient was calculated by ProtParam tool http://www.expasy.ch: 94 240 M^-1 ^cm^-1^.

### RM.Eco29kI REase- and MTase reaction optima

Optimum conditions for endonuclease and methyltransferase reactions of RM.Eco29kI were studied as before [14,15].

### Investigation of AdoMet influence on REase activity of RM.Eco29kI

To study the AdoMet effect on REase activity of RM.Eco29kI, 0.5 μg of phage φ80vir DNA was treated with 2-fold dilutions of 0.1 μg RM.Eco29kI at REase (50 mM NaCl, 10 mM MgCl_2_; 20 mM Tris-HCl, pH 7.5) and MTase (50 mM NaCl; 5 mM β-mercaptoethanol; 20 mM Tris-HCl, pH 8.0, with 10 mM MgCl_2_) optimal conditions with or without excess AdoMet (10 μM) for 1 h at 37°C. Reaction products were analyzed electrophoretically in 0.8% agarose gel.

### Unit definition

The amount of enzyme required to transfer 1 pmol of (^3^H)-methyl groups to DNA per minute with saturating concentrations of substrates at 37°C has been taken as 1 AU of the RM.Eco29kI DNA methyltransferase activity. The amount of enzyme required to cut 1 μg of phage φ80vir DNA during 1 h at 37°C has been taken as 1 AU of the RM.Eco29kI restriction endonuclease activity. Both reactions were carried out under predefined optimal reaction conditions.

## List of abbreviations used

ORF: open reading frame; REase: restriction endonuclease; MTase: methyltransferase; RMS: restriction-modification system; AdoMet: S-adenosyl-L-methionine; TRD: target recognition domain; R.Eco29kI: REase Eco29kI; M.Eco29kI: MTase Eco29kI; RM.Eco29kI: fused REase and MTase Eco29kI in one polypeptide; Aa: amino acids; bp: base pairs; 6His: 6 Histidine residues; LB medium: Luria-Bertani medium; Rpm: rotations per minute; EDTA: ethylenediaminetetraacetic acid; DTT: dithiothreitol; PAGE: polyacrylamide gel electrophoresis; AU: arbitrary units; pMol: 10^-12 ^Mol; μg: 10^-6 ^g.

## Authors' contributions

DN and AS designed experiments; MM and DN performed experiments; DN wrote the paper. MM, AS and DN read and approved the final manuscript.
